# Exploring the Comprehensive Neuroprotective and Anticancer Potential of Afzelin

**DOI:** 10.3390/ph17060701

**Published:** 2024-05-28

**Authors:** Mateusz Kciuk, Nitika Garg, Sanchit Dhankhar, Monika Saini, Somdutt Mujwar, Sushma Devi, Samrat Chauhan, Thakur Gurjeet Singh, Randhir Singh, Beata Marciniak, Adrianna Gielecińska, Renata Kontek

**Affiliations:** 1 Department of Molecular Biotechnology and Genetics, Faculty of Biology and Environmental Protection, University of Lodz, 12/16 Banacha St., 90-237 Lodz, Poland; mateusz.kciuk@biol.uni.lodz.pl (M.K.); beata.marciniak@biol.uni.lodz.pl (B.M.); adrianna.gielecinska@edu.uni.lodz.pl (A.G.); renata.kontek@biol.uni.lodz.pl (R.K.); 2 Chitkara College of Pharmacy, Chitkara University, Rajpura 140401, Punjab, India; nitikagarg1609@gmail.com (N.G.); sanchitdhankhar@gmail.com (S.D.); sushma.mehla@gmail.com (S.D.); gurjeet.singh@chitkara.edu.in (T.G.S.); 3 M. M. College of Pharmacy, Maharishi Markandeshwar (Deemed to be) University, Mullana, Ambala 133207, Haryana, India; monika210692@gmail.com; 4 Swami Vivekanand College of Pharmacy, Ramnagar, Banur 140601, Punjab, India; 5 Department of Pharmacology, Central University of Punjab, Bathinda 151401, Punjab, India; randhirsingh.dahiya@gmail.com; 6 Doctoral School of Exact and Natural Sciences, University of Lodz, 90-237 Lodz, Poland

**Keywords:** afzelin, neurodegeneration, neuroinflammation, Alzheimer’s disease, Parkinson’s disease, cancer

## Abstract

Neurodegenerative diseases (Alzheimer’s disease, Parkinson’s disease, Huntington’s disease, and others) and cancer, seemingly disparate in their etiology and manifestation, exhibit intriguing associations in certain cellular and molecular processes. Both cancer and neurodegenerative diseases involve the deregulation of cellular processes such as apoptosis, proliferation, and DNA repair and pose a significant global health challenge. Afzelin (kaempferol 3-O-rhamnoside) is a flavonoid compound abundant in various plant sources. Afzelin exhibits a diverse range of biological activities, offering promising prospects for the treatment of diseases hallmarked by oxidative stress and deregulation of cell death pathways. Its protective potential against oxidative stress is also promising for alleviating the side effects of chemotherapy. This review explores the potential therapeutic implications of afzelin, including its capacity to mitigate oxidative stress, modulate inflammation, and promote cellular regeneration in neurodegenerative and cancer diseases.

## 1. Introduction

Neurodegenerative diseases are complex conditions that share the characteristics of cell death and malfunctioning of neurons. These conditions, sometimes referred to as geriatric diseases, are primarily characterized by a gradual degeneration of neurons, ultimately leading to the decline of crucial cognitive, motor, and sensory skills [1,2,3]. The defining features of Alzheimer’s disease, a prevalent neurodegenerative disorder, involve the development of aberrant protein aggregates in the brain, including beta-amyloid plaques and tau tangles. The presence of these aggregates hinders the transmission of brain signals and induces an inflammatory response, both of which are implicated in the development of cognitive dysfunction [4,5]. Similarly, Parkinson’s disease is defined by the degeneration of dopaminergic neurons in the substantia nigra, which ultimately leads to the death of these neurons. The depletion of neurons leads to motor dysfunctions characterized by tremors, rigidity, and bradykinesia [6]. In contrast, Huntington’s disease arises as a consequence of a genetic mutation that results in the misfolding of proteins. This phenomenon results in a progressive deterioration of cognitive and physical functions. The neuronal cell loss implicated in amyotrophic lateral sclerosis (ALS), colloquially referred to as Lou Gehrig’s disease, is accountable for muscle atrophy, culminating in eventual paralysis [7]. Various types of neurodegeneration, including multiple sclerosis (MS) [8] and frontotemporal dementia (FTD) [9], are characterized by specific patterns of neuronal degeneration and exhibit diverse manifestations across the brain and spinal cord [10]. Despite their intended objectives of symptom relief and disease progression cessation, conventional therapies often fail to provide comprehensive solutions. The examination of diverse natural compounds has arisen as a feasible avenue to harness the potential of nature’s abundant medicinal properties to treat these intricate disorders. Natural chemicals are derived from diverse sources, encompassing plants, herbs, fungi, and marine life [11]. Within the research area of neurodegenerative disorder treatments, some naturally occurring compounds have garnered interest because of their inherent neuroprotective, anti-inflammatory, antioxidative, and modulatory properties. For example, curcumin (a compound extracted from turmeric), resveratrol (derived from red grapes), and flavonoids (isolated from citrus fruits) have all demonstrated neuroprotective properties in various studies. These compounds are believed to provide neuroprotection due to their impact on inflammation and toxic protein aggregation-induced neuronal injury. Furthermore, it was shown that naturally derived compounds possess the capacity to regulate cellular pathways that play a critical role in the survival and proliferation of cells [12,13,14]. 

The increasing prevalence of cancer and the limitations of conventional therapies have spurred a growing interest in exploring alternative treatment modalities, particularly the use of natural products. Bioactive compounds such as polyphenols, alkaloids, terpenoids, and flavonoids have exhibited potent anti-proliferative, anti-angiogenic, and pro-apoptotic effects across a spectrum of cancer types. Moreover, the potential synergistic interactions between natural products and conventional anti-cancer therapies are explored. Evidence suggests that natural products can enhance the efficacy of chemotherapy and radiotherapy while mitigating their adverse effects. The modulation of drug resistance mechanisms and the promotion of sensitization to standard therapies underscore the promising role of natural products in combination regimens [15].

Oxidative stress, which is caused by an imbalance between reactive oxygen species (ROS) and antioxidants, is also an essential factor in the pathophysiology of neurodegenerative diseases [16,17,18,19] and cancer [20,21,22]. Natural compounds, such as vitamin E [23,24], vitamin C [25,26], or flavonoids, can scavenge dangerous free radicals, thereby minimizing the amount of oxidative damage that is caused to neurons. This antioxidative capability goes beyond only protecting cells; it also has the potential to influence the signaling cascades and gene expression inside the cells [27,28]. 

**Figure 1:**
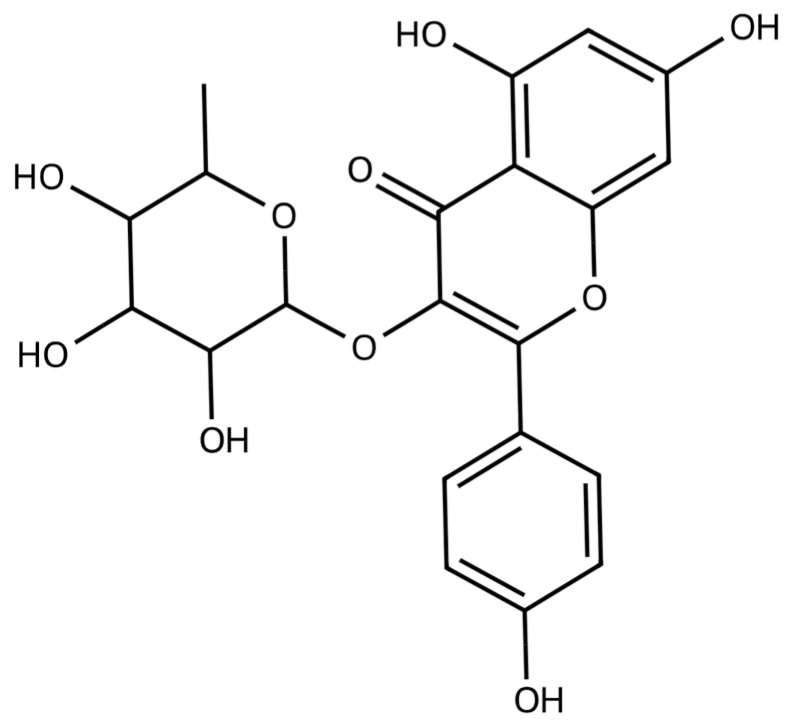
Chemical structure of kaempferol 3-O-rhamnoside (Afzelin).

Flavonoids represent a class of polyphenolic compounds that are widely distributed across the plant kingdom. Afzelin (Figure 1), also known as kaempferol 3-O-rhamnoside, is a flavonoid compound that can be found in high concentrations in a wide variety of plant sources. The diverse range of biological activities, including the antioxidative properties exhibited by afzelin, holds a significant translational potential of this compound in the prevention and therapy of cancer and neurodegenerative diseases manifested by oxidative stress and deregulation of cell death pathways [29,30,31,32]. Moreover, intrinsic links have been discovered between oxidative stress and inflammation. Therefore, the ROS-scavenging effects of afzelin could mitigate neuroinflammation, which constitutes a characteristic feature of neurodegenerative disorders such as Alzheimer’s disease and Parkinson’s disease [33,34]. 

## 2. Sources and Pharmacological Profile of Afzelin

The anti-bacterial, anti-inflammatory, antioxidant, and cytoprotective effects of afzelin have been supported by numerous studies [29,30,31,32].

Afzelin come from a variety of different places, including but not limited to the following:

*Houttuynia cordata:* The plant under consideration is native to Southeast Asia and possesses a rich historical background in its utilization within traditional medicinal practices. Its applications encompass a diverse array of diseases, such as cancer, inflammation, infection, and fever. The main flavonoids found in the *Houttuynia cordata* plant include afzelin, quercetin, and hyperoside [35,36]. 

*Nymphaea odorata*: The plant is native to North America and is alternatively referred to as the American white waterlily. This botanical remedy has a long-standing medicinal history, as it was historically used for the treatment of skin conditions, wounds, inflammation, and cancer. Afzelin, kaempferol, quercetin, myricetin, and gallic acid represent abundant phenolic compounds found in this plant [37,38]. 

*Kalanchoe pinnata:* This plant is commonly known as an ‘air plant’ or ‘miracle leaf’ and is native to regions in Africa and Asia. The *Kalanchoe pinnata* plant has been employed in traditional medicinal practices for the treatment of various diseases, including ulcers, asthma, coughing, and diarrhea. Afzelin represents one of the flavonoids present in this plant. Others include quercitrin, quercetin, kaempferol, and rutin. The hepatoprotective benefits of afzelin, derived from *Kalanchoe pinnata*, have been demonstrated, indicating its potential to mitigate liver damage induced by carbon tetrachloride [39,40,41,42].

*Quercus* spp. (Oak Trees): Oak trees are renowned for their impressive stature in woodland areas and natural environments. Additionally, they offer a wide array of biologically active secondary metabolites, including afzelin. Afzelin is present in several anatomical components of oak trees, encompassing their foliage, bark, and reproductive structures, such as acorns. *Quercus* species exhibit a wide range of climatic and geographical distributions. Variability in the concentration of afzelin in *Quercus* plants can be attributed to several factors, including species differences, the conditions under which the plant is growing, and the age of the plant [43,44].

*Filipendula ulmaria* (Meadowsweet): This perennial herbaceous plant is native to both Europe and Asia. It has a rich history of application in the practice of herbal medicine. Afzelin found in meadowsweet is responsible for the pain-relieving and anti-inflammatory effects of the herb [45]. 

*Hawthorn* (*Crataegus* spp.): This plant belongs to the *Rosaceae* family and is well-known for its beneficial effects on the cardiovascular system. The antioxidative and vasorelaxant properties of hawthorn can be partially attributed to the presence of afzelin in the plant [46,47]. 

**Figure 2:**
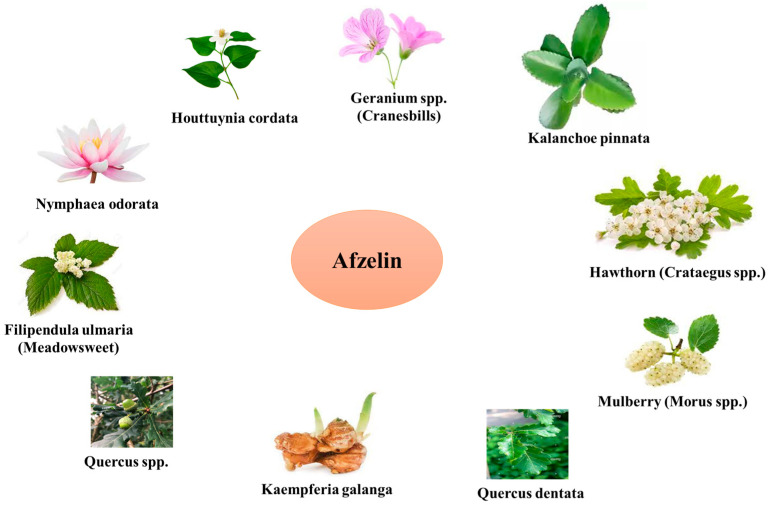
Natural sources of afzelin.

The natural sources of afzelin were summarized in Figure 2.

## 3. Biological Activity of Afzelin

### 3.1. Antioxidative Effects of Afzelin

Oxidative stress arises from a state of disequilibrium between the generation and buildup of ROS within cells and tissues, as well as the capacity of a biological system to effectively neutralize these reactive byproducts. ROS fulfill various physiological functions, such as cell signaling, and are typically produced as byproducts of oxygen metabolism. However, exposure to environmental stressors like UV radiation, ionizing radiation, pollutants, and heavy metals, as well as the presence of xenobiotics, significantly enhance ROS production. Consequently, this imbalance results in oxidative stress, leading to cellular and tissue damage [48]. Afzelin’s potential therapeutic applications in the context of neurodegenerative illnesses and cancer could be mainly attributed to the chemical’s ability to alter redox signaling pathways and prevent oxidative stress [30,49,50,51] through the following:

- Scavenging of free radicals: Highly reactive chemicals, such as free radicals and ROS, can oxidatively damage biological components like DNA, proteins, and lipids [52]. Afzelin has been found to possess ROS-scavenging characteristics, suggesting it can neutralize the effects of dangerous compounds through interaction with them [53,54].
- Metal chelation: Afzelin also possesses metal chelation capabilities, making it possible for it to bind to metal ions such as those of iron and copper. These metal ions are well-known to have a role in the generation of free radicals via the Fenton and Haber-Weiss reactions. Afzelin is effective in blocking the generation of ROS because of its binding affinity for these metals [55,56,57].
- Activation of nuclear factor erythroid 2-related factor 2 (NRF2) pathway: The NRF2 pathway plays a significant role in the regulation of the antioxidant response in cells via transcriptional control of many target genes involved in the maintenance of oxidation-reduction homeostasis inside the cell. The antioxidative actions of kaempferol and its derivatives, including afzelin, extend to the protection of cellular components like cell membranes and mitochondria from the destructive effects of oxidation. Thereby, afzelin may contribute to the maintenance of proper cellular function by preserving the structural integrity of these components [58].

Mitochondria are crucial for the various biochemical activities that are fundamental to the survival of cells. The morphology of mitochondria undergoes alterations in response to external stimuli and metabolic fluctuations through the processes of fission and fusion, collectively known as mitochondrial dynamics. The elimination of impaired mitochondria is facilitated through a cellular mechanism referred to as mitophagy, wherein these damaged organelles undergo breakdown via a distinct autophagosomal pathway. In recent years, significant endeavors have been undertaken to examine the influence of mitochondrial dysfunction manifested by excessive production of ROS, disturbances in mitochondrial calcium homeostasis, depletion of adenosine triphosphate (ATP), impairments in mitochondrial dynamics and transport, and deficiencies in mitophagy on the development of Alzheimer’s disease [59,60], Parkinson’s disease [61,62], other neurodegenerative diseases [63], and cancer [64,65].

Lee et al. investigated the cytoprotective mechanisms of afzelin against D-galactosamine (GalN)/lipopolysaccharide (LPS)-induced fulminant hepatic failure (FHF), with a specific emphasis on mitochondrial quality control and dynamics. The findings of the study demonstrated that the administration of the flavonoid compound resulted in enhanced survival rates and decreased levels of alanine aminotransferase and pro-inflammatory cytokines in mice that were treated with GalN/LPS. Afzelin contributed to a reduction in mitochondrial damage in mice treated with GalN/LPS, as evidenced by a decrease in mitochondrial swelling and a reduction in the mitochondrial glutamate dehydrogenase activity. The administration of afzelin resulted in an augmentation of mitochondrial biogenesis, as evidenced by elevated levels of PPAR-γ coactivator 1α (PGC-1α), nuclear respiratory factor 1 (NRF1), and mitochondrial transcription factor A (TFAM) [31]. 

PGC-1α has been shown to exert a protective effect on neuroblastoma cells by mitigating neuronal mortality and neuroinflammation produced by amyloid-beta (Aβ). Additionally, the neuroprotective impact of PGC-1α is governed by the nuclear factor kappa B (NF-κB) pathway [66]. There is evidence to suggest that PGC-1α plays a crucial role in the preservation of neuronal viability and the facilitation of synaptic transmission. Changes in the levels of PGC-1α expression were observed in patients with neurodegenerative disorders, including Alzheimer’s [67]. The down-regulation of PGC-1α in various brain regions, such as the hippocampus, substantia nigra (SN), cortex, striatum, and spinal cord, has been observed in animal and cellular models of neurodegenerative diseases. This down-regulation is associated with neurological damage, including oxidative stress, neuronal loss, and motor disorders. The existing body of evidence suggests that the overexpression of PGC-1α may offer a promising therapeutic strategy in mitigating the onset and advancement of neuronal damage [68]. Changes and adjustments in cellular metabolism are distinctive characteristics of cancer cells; therefore, it is unsurprising that PGC-1α has a function in cancer. Recent papers have demonstrated that the expression of PGC-1α is changed in metastatic tumors because of alterations in cellular metabolism, as recently reviewed [69].

TFAM is an important protein for genome maintenance as it binds to and stabilizes mitochondrial DNA (mtDNA). Multiple neurodegenerative diseases exhibit alterations in mtDNA and TFAM protein levels. In studies of neurodegenerative models, it has been observed that the levels of mtDNA exhibit a reduction of approximately 30%. In both cellular and animal models of neurodegeneration, the overexpression of TFAM and the administration of TFAM replacement therapy demonstrated significantly enhanced brain function and composition [70].

Furthermore, afzelin use was associated with the reduction in the expression of mitophagy-associated proteins, namely parkin (PARKIN) and PTEN-induced putative kinase 1 (PINK1) [31]. The signaling pathways involving PINK1 and PARKIN are crucial in regulating mitophagy. PINK1 exhibits an accumulation at the outer mitochondrial membrane (OMM) as a result of a decrease in mitochondrial membrane potential (ΔΨm) due to injury or malfunction. Consequently, this process induces the translocation of PARKIN from the cytosol to the OMM, where its E3 enzymatic activity facilitates mitophagy by ubiquitinating mitochondrial proteins, ultimately resulting in the degeneration of mitochondria. Neurodegenerative disorders such as Alzheimer’s disease and Parkinson’s disease [71] or cancer [72,73] often exhibit the presence of defective mitophagy and aberrant PINK1/PARKIN signaling pathways. In addition, it was observed that the administration of GalN/LPS resulted in a substantial elevation in the expression of dynamin-related protein 1 (DNM1L), a protein associated with fission processes, while concurrently causing a reduction in the expression of mitofusin 2 (MFN2), a protein involved in fusion processes. However, the impact of GalN/LPS on these protein levels was mitigated by the presence of afzelin [31].

It has been shown that kaempferol-3-O-β-d-glucuronate or afzelin inhibits neuroinflammation by preventing the phosphorylation of mitogen-activated protein kinases (MAPKs) [74,75] and also boosts antioxidant defenses by increasing the activity of the NRF2/heme oxygenase-1 (HO-1) signaling pathway possibly via glycogen synthase kinase-3β (GSK-3β) inhibition [50]. NRF2 constitutes a pivotal transcription factor that is of significant importance in the cellular response to oxidative stress and the maintenance of redox equilibrium in cells. The fundamental role of this NRF2 is to control the activation of several antioxidant and detoxification proteins in reaction to oxidative stress and other stressors present in the environment. NRF2 facilitates the transcriptional activation of a diverse array of genes that encode antioxidant enzymes, phase II detoxification enzymes, and other proteins that safeguard cellular integrity. The genes encompassed in this set include HO-1, NAD(P)H quinone oxidoreductase 1 (NQO1), glutathione S-transferases (GSTs), and superoxide dismutase (SOD). Several studies have shown that activation of the NRF2 signaling pathway can protect neuronal cells against oxidative stress, as reviewed by many authors [76,77,78], and its activation could constitute an important approach to the treatment of Alzheimer’s disease [79], Parkinson’s disease [78], ALS [80], and other movement disorders [81]. On the other hand, the NRF2 signaling pathway can act as a double-edged sword in cancer. The NRF2 pathway protects against exogenous chemical-induced oxidative damage. It regulates downstream cytoprotective genes to preserve redox equilibrium and has anti-inflammatory and anti-cancer actions, ensuring cell survival. Aberrant NRF2 activity is related to poor prognosis in cancer patients. Constitutive NRF2 activation in malignancies increases cancer cell proliferation by metabolic reprogramming, suppression of apoptosis, and enhanced self-renewal potential of cancer stem cells. Furthermore, NRF2 is linked to cancer cell chemoresistance and radioresistance [82]. HO-1 is an enzyme that performs a pivotal function in the process of heme catabolism, which entails the degradation of heme into biliverdin, carbon monoxide (CO), and iron [83]. HO-1 is a member of the heme oxygenase enzyme family, alongside another isoform—heme oxygenase-2 (HO-2). However, HO-2 differs from HO-1 in terms of its tissue distribution and regulatory function [84]. Biliverdin undergoes conversion into bilirubin by the catalytic action of an enzyme known as biliverdin reductase. Bilirubin possesses antioxidant capabilities that contribute to cellular protection against oxidative stress through its ability to scavenge free radicals [85]. HO-1 also plays a crucial role in the modulation of the immune response and the regulation of inflammation. It can inhibit the synthesis of pro-inflammatory molecules and enhance the synthesis of anti-inflammatory cytokines, thereby facilitating the mitigation of inflammation [86,87]. The phenomenon of HO-1 overexpression has been linked to the recovery of numerous ailments, such as cardiovascular diseases, neurodegenerative disorders, and different inflammatory conditions. The stimulation of HO-1 activity has the potential to ameliorate tissue damage and facilitate tissue regeneration. The potential therapeutic targeting of HO-1 and its associated targets have been extensively investigated due to their protective and anti-inflammatory effects across a range of disorders. Efforts are currently undertaken to pharmacologically stimulate the expression of HO-1 or regulate its functionality as a potential therapeutic approach for illnesses that are characterized by the presence of oxidative stress and inflammation [88,89,90,91]. 

#### Anti-Inflammatory Effects of Afzelin

Neuroinflammation, a complex immune response that occurs within the central nervous system (CNS), is an underlying factor that is shared by many of these conditions. Neuroinflammation plays a significant role in neuronal damage and the unrelenting progression of these diseases when it is dysregulated. The activation of glial cells, namely microglia and astrocytes, in pathological states such as Alzheimer’s disease, Parkinson’s disease, and MS initiates an intricate cascade of events leading to the secretion of pro-inflammatory mediators, cytokines, chemokines, and ROS. The aberrant immune response contributes to the perpetuation of neurotoxicity and intensifies neuronal cell death. This topic was covered in excellent review papers by multiple authors and will not be discussed here in detail [92,93,94,95,96,97]. Chronic inflammation has emerged as a pivotal contributor to cancer initiation, progression, and metastasis, representing a dynamic and intricate interplay between the immune system and tumorigenesis. Inflammatory responses, intended as protective mechanisms against infections or tissue damage, can become persistently activated, fostering a microenvironment conducive to carcinogenesis [98]. 

**Figure 3:**
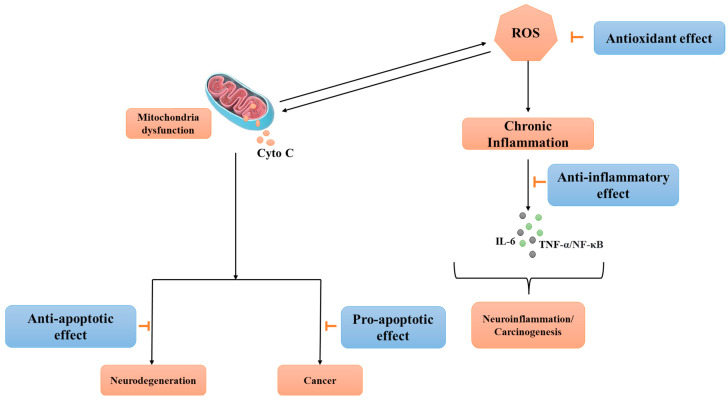
Antioxidative, anti-inflammatory, and anti/pro-apoptotic effects of afzelin.

Focusing on afzelin, its anti-inflammatory properties are believed to stem from its interactions at the molecular level with several cellular processes and components associated with the inflammatory response [30,35,99]. Elevated concentrations of nitric oxide (NO), mostly generated by the synthesis of inducible nitric oxide synthase (iNOS) in glial cells, have been discovered to induce neuronal cell death by inhibiting neuronal mitochondrial cytochrome oxidase. Therefore, targeting NO signaling with natural compounds can constitute a feasible approach. Afzelin was shown to act as an inhibitor of NO production with an IC_50_ value of 42.8 µg/mL with comparable efficacy to nitric oxide inhibitor L-NMMA used in the experiment as a positive control (IC_50_ of 42.1 µg/mL) [100]. Furthermore, interleukin-1 beta (IL-1), interleukin-6 (IL-6), and tumor necrosis factor-alpha (TNF-α) production were shown to be down-regulated by afzelin treatment suggesting that this compound can dampen the inflammation in the human skin for effective prevention of skin cancers [101,102]. The administration of flavonoids, specifically quercitrin, isoquercitrin, and afzelin, to human neuroblastoma SH-SY5Y cells have demonstrated advantageous outcomes via modulating inflammation, apoptosis (through inhibition of caspase activation), and ROS-scavenging [103,104]. In contrast, the pro-apoptotic properties of afzelin in cancer cells may account for its anti-cancer effects [105,106], as described in the following sections of this work (Figure 3).

### 3.2. Neuroprotective and Neurogenic Effects

Brain-derived neurotrophic factor (BDNF) is a member of the neurotrophin protein family and works as a pivotal growth factor required for the integrity, individuality, and adaptability of neuronal populations [107]. BDNF is broadly expressed in the mammalian brain, with especially high levels in the hippocampus and parahippocampal regions, both of which are involved in memory formation. Its expression is modulated dynamically in response to external cues and internal states, which at the molecular level involves the cooperation of multiple events, including transcriptional control, epigenetic processes, and post-translational modifications. BDNF interacts with its receptors, tropomyosin kinase B (TRKB) and P75 neurotrophin receptor (P75NTR), to play an important role in synaptic transmission and activity-dependent plasticity [108]. By interacting with these proteins, BDNF triggers a cascade of signaling events that control gene expression, synaptic strength, neuronal shape, and neurogenesis. In addition, BDNF is involved in a wide variety of physiological and pathological processes, including nutritional control, stress response, aging, neurodegeneration, and neuropsychiatric diseases. Changes in BDNF expression levels are associated with Alzheimer’s disease, Parkinson’s disease, Huntington’s disease, and other forms of neurodegeneration [109,110]. As a consequence of this, BDNF is being investigated as a potential therapeutic target for neurological conditions. An increase in the synthesis of BDNF has been suggested as a therapeutic avenue for neurodegenerative disease treatment [109,111]. 

The introduction of afzelin into the mice brains on a chronic schedule resulted in the improvement of synaptic plasticity and cognitive/memory functions in animals that were administered scopolamine. Research conducted on the hippocampi of mice has demonstrated that the restorative effects observed in the cholinergic systems and molecular signal transduction through cyclic AMP response element-binding protein (CREB)-BDNF pathways can be attributed to the response elicited by afzelin. It was demonstrated that the administration of afzelin resulted in enhanced neurocognitive and neuroprotective effects on synaptic plasticity and behaviors, partially attributed to the up-regulation of CREB-BDNF signaling [112]. Moreover, the increased BDNF production may promote neuroprotective activity in hippocampal neurons through the stimulation of phosphatidylinositol 3-kinase (PI3K) and RAS/MAPK pathways and consequent abrogation of apoptotic cell death as evidenced by reduced caspase activity and increased B-cell lymphoma 2 (BCL2) protein expression [113]

Aldose reductase (AR) is an enzymatic protein responsible for the conversion of glucose into sorbitol within the NADPH-dependent polyol pathway of glucose metabolism. AR plays a pivotal role in the activation of microglia and the progression of retinal neurodegeneration. The activation of surveillance microglia occurs with the application or deposition of β-amyloid, subsequently leading to the formation of ROS, increased release of TNF-α, and enhanced phagocytosis. These processes collectively contribute to the death of neurons. The enhanced migratory capacity of microglia may result in cerebral injury. The inhibition of AR activity has the potential to impede the activation and migration of microglia, hence offering a potential therapeutic strategy for mitigating neuronal cell death and decelerating the progression of Alzheimer’s disease [114]. Afzelin was shown to act as an AR inhibitor with an IC_50_ concentration of 1.91 μM compared with 12.87 μM estimated for kaempferol and could constitute one of the underlying causes of down-regulation of inflammatory mediators expression observed during the study [115], as shown for other AR inhibitors. These effects can be attributed to the modulation of MAPK and NF-κB-mediated pathways [116]. 

The areas inside the brain that are responsible for the production of significant quantities of neuromelanin (NM), a dark cytoplasmic pigment, are limited to the SN and locus coeruleus (LC). Evidence suggests that NM serves an initial protective function by isolating toxic catecholamine metabolites and heavy metals. However, it is posited that this protective role may transition into a destructive one during the aging process, especially in the context of Parkinson’s disease. This transition occurs as the aforementioned toxic substances accumulate to a point where they overpower cellular recovery machinery and are subsequently released during the process of neurodegeneration. The accumulation of NM was linked to the occurrence of neurodegeneration, as well as a strong neuroinflammatory response [117]. The process of synthesizing human NM is considered to be analogous to the generation of melanin in melanocytes. In the skin, melanin synthesis occurs through the conversion of DOPAquinone (DQ) by the enzyme tyrosinase. On the other hand, NM synthesis in neurons takes place through the conversion of dopamine quinone (DAQ) by the enzymes tyrosine hydroxylase (TH) and aromatic L-amino acid decarboxylase (AADC). Dopamine within the cytoplasm exhibits a high level of reactivity and is thought to undergo spontaneous oxidation or be oxidized by an undiscovered tyrosinase enzyme, resulting in the formation of DAQ. Subsequently, DAQ is converted into NM. The buildup of intracellular NM over a certain threshold has been documented to be linked to the death of dopaminergic neurons and the manifestation of symptoms associated with Parkinson’s disease. Therefore, tyrosinase inhibition could constitute a potential therapeutic approach in this neurodegenerative disease [118]. 

Rho et al. aimed to assess the tyrosinase inhibitory activity of the ethanolic extract derived from the leaves of kenaf (*Hibiscus cannabinus* L.) both before and after exposure to far-infrared (FIR) irradiation. No inhibitory activity of the extract was seen in a tyrosinase enzymatic activity experiment before FIR irradiation. Nevertheless, when subjecting it to FIR, a noteworthy degree of tyrosinase inhibitory activity (IC_50_ = 3500 ppm) was detected. The high-performance liquid chromatography (HPLC) examination revealed the presence of derhamnosylation products, specifically kaempferol, afzelin, and α-rhamnoisorobin. The observed inhibitory activity could perhaps be attributed to the presence of derhamnosylation products [119].

Afzelin is one of 28 metabolites revealed by liquid chromatography–tandem mass spectrometry (LC-MS/MS) in the ethanolic extract of *Polygonum minus*. Studies of neuroprotection against H_2_O_2_-induced oxidative stress performed on differentiated SH-SY5Y cells revealed activation of NRF2/antioxidant response element (ARE), NF-κB/IκB, and MAPK signaling pathways. A 48 h pre-incubation of cells with PMEE extract resulted in a significant up-regulation of *NRF2*, *NQO1*, *SOD1*, *SOD2*, and catalase gene expression levels compared to both PMEE extract and H_2_O_2_ treatments. A similar effect was observed for *MAPK8* (JNK), *MAPK14* (p38), and *PPP2CA* (PP2A) genes. Also, there are various in silico and molecular docking studies that showed that afzelin was one of the compounds present in the *Polygonum minus* extract with the highest stability and docking score [120].

Acetylcholine (ACH) is a neurotransmitter that may play a positive role in modulating the cellular response to oxidative stress by regulating antioxidant processes, mitochondrial function, and protection against negative effects resulting from excess ROS. In some neurodegenerative diseases like Alzheimer’s disease, low levels of ACH are observed. Acetylcholinesterase (AChE) is an enzyme responsible for metabolizing the ACH into acetic acid and choline, which allow for the termination of the signal transmission at the synapses. AChE inhibitors have beneficial effects in certain neurological conditions by temporarily enhancing cholinergic neurotransmission [121]. Under conditions of H_2_O_2_-induced oxidative stress, prior administration of PMEE increased ACH concentration in differentiated SH-SY5Y cells supernatant. Molecular docking results showed that afzelin was one of the three compounds present in the *Polygonum minus* ethanolic extract with the highest docking score to AChE [120]. 

### 3.3. Anti-Cancer Effects of Afzelin

Flavonoids, which include flavonol glycoside, have long been studied for their anti-cancer effects. In the case of afzelin, recent years have yielded the results of the few studies to date that indicate the potential to induce cell death and inhibit tumor proliferation in an in vitro model. Androgen-sensitive LNCaP and androgen-independent PC-3 cell lines were used in one of the anti-cancer activity studies on afzelin. The investigation revealed that afzelin, at a concentration of 1 µg/mL, caused a significant decrease in prostate cancer cell proliferation after 24 h incubation. Flow cytometry cell cycle evaluation showed accumulation of PC-3 and LNCaP cells in the G_0_ phase (27.6% and 19.4% of the population, respectively) after 10 µg/mL afzelin exposure and indicated the occurrence of extensive apoptosis or an ongoing cytotoxic response. At the molecular level, afzelin suppressed Rho GTPase family proteins such as LIM domain kinase 1 (LIMK1), myotonic dystrophy kinase-related Cdc42-binding kinase α (MRCKα), and Rho-associated coiled-coil-containing protein kinase (ROCK1) expression. The Rho GTPase family is intricately linked to the modulation of actin cytoskeleton arrangement, mediation of the focal adhesions, stress fiber formation, and regulation of cell proliferation and differentiation via activation of different proteins. Expression of LIMK1 is observed to be elevated in prostate cancer cells, while ROCK1 and MRCKα are involved in its activation. In examined prostate cancer cells, a reduction in the phosphorylation level of the aforementioned proteins was detected dependent on the concentration of afzelin tested [38].

Suppression of cell migration of highly invasive MDA-MB-231 triple-negative breast cancer (TNBC) cells following afzelin treatment was shown to be mediated by focal adhesion kinase (FAK) and Ras-related C3 botulinum toxin substrate 1 (RAC1) deregulation. FAK and RAC1 proteins interact in signaling pathways that control the organization of the cytoskeleton and the dynamics of focal adhesions, which in turn affect cell migration processes. Afzelin induced moderate cytotoxicity of MDA-MB-231 cells (IC_50_ = 992 μg/mL); however, at the same time, it actively reduced focal adhesion formation in a concentration-dependent manner. Because FAK and RAC1 are often excessively activated in numerous tumor types, they increase the likelihood of invasion and metastasis, which correlates with a worse prognosis for patients. Afzelin at 400 and 800 µg/mL significantly reduced RAC1 GTPase activation by 60% compared to the negative control, while the highest concentration also reduced FAK and phosphorylated FAK (pFAK) expression [122]. Additionally, Rachimi et al., using the reverse docking method, confirmed the inhibitory potential of afzelin toward molecular targets relevant to TNBC, including FAK, ERK2, and KRAS [123].

The pro-apoptotic efficacy of afzelin has been assessed in vitro in a variety of cancer cell types. For instance, kaempferol-3-O-rhamnoside isolated from leaves of *Schima wallichii* Korth was able to reduce the proliferation of MCF-7 human breast cancer cells. The findings demonstrated that afzelin triggers apoptotic mechanisms through activation of the caspase signaling pathway [124]. Afzelin was found to induce apoptotic cell death in gastric cancer cells (AGS) [105] as well as lung cancer cells (A549 and H1299) [106]. In the case of A549 cells, exposure to 60 and 120 µM afzelin resulted in an increase in the mRNA levels (an increase of 30% and 80%, respectively) of the pro-apoptotic factor BAX in comparison with non-treated cells. Increased afzelin concentrations resulted in stimulation of the caspase cascade, with the involvement of both caspase 8 and caspase 9 [105]. In a study using a model of lung cancer, characteristic indicators of immunogenic cell death, such as calreticulin exposure or high-mobility group box 1 (HMGB1) and ATP release, were observed [106]. 

## 4. ADMET Properties of Afzelin 

Afzelin was shown to reach a maximal concentration of 211.02 ± 27.97 μg/L at a t_max_ = 0.83 ± 0.26 h, following administration of the inflorescence of *Polygonum orientale* at a concentration of 0.3 g/mL, which is comparable to a crude herb dosage of 86 g/kg or an extract dosage of 3 g/kg. This dosage constituted six times the recommended daily dose for an adult [125].

The study conducted by Alves et al. provided evidence indicating that hydroalcoholic extract of *Copaifera lansdorffii* Desf. (CLE) did not exhibit genotoxic effects in the mouse micronucleus experiment. The administration of CLE plus doxorubicin to animals resulted in a considerable reduction in the number of micronuclei, as compared to animals that were treated only with doxorubicin [126]. The protective effects of afzelin and quercitrin against the cytotoxicity induced by doxorubicin or methyl methanesulfonate (MMS) in V79 and HepG2 cell lines were investigated using the same extract. Moreover, it was observed that CLE did not elicit a statistically significant alteration in the occurrence of chromosomal abnormalities and micronuclei in the tested cells. Additionally, the number of revertants in the Ames test remained the same, indicating the absence of its mutagenic effects. On the other hand, it was observed that CLE exhibited anti-genotoxic properties in mammalian cells. The administration of afzelin and quercitrin showed a reduction in genotoxicity generated by MMS in HepG2 cells. The observed antigenotoxic impact of CLE in this study could potentially be ascribed, at least in part, to the antioxidative activity of the combined main components, namely afzelin and quercitrin [127].

Afzelin is widely recognized for its antioxidative characteristics [128], which can be seen from its capacity to scavenge various oxidative species. Despite its beneficial properties, it was found to induce cytotoxic effects that can be beneficial for the treatment of cancer. Afzelin was found to have a promotional effect on neutrophil death, particularly when the oxidative burst was induced by phorbol myristate acetate. Hence, while its widely recognized ability to scavenge free radicals and oxidants, this molecule can inflict side effects in living organisms by exerting its effects on erythrocytes and neutrophils [129].

Given the importance of assessing other toxicological endpoints for new promising molecules like afzelin, future research could focus on expanding the evaluation of its ADMET properties beyond the scope of its genotoxicity and cytotoxicity. Investigating the metabolic fate of pure afzelin in vivo could provide insights into its biotransformation pathways and the formation of metabolites. This information is crucial for understanding its pharmacokinetic profile and potential interactions with other drugs or compounds. Assessing the distribution of afzelin in various tissues after administration could help determine its target organs and potential accumulation in specific tissues. Understanding its tissue distribution profile is essential for predicting its efficacy and potential toxicity. Studying the excretion pathways of afzelin, including renal and biliary excretion, could provide valuable information about its elimination kinetics and potential for accumulation. This knowledge is important for determining appropriate dosing regimens and potential risks associated with long-term exposure. Conducting comprehensive toxicity studies to evaluate the acute and chronic effects of afzelin at various doses and duration of exposure in animal models could help assess its safety profile. This includes assessing its effects on organ function, histopathological changes, and overall systemic toxicity. In summary, by systematically evaluating these additional toxicological endpoints, researchers could gain a more comprehensive understanding of the safety profile of afzelin, which is essential for its further development as a potential therapeutic agent.

## 5. Conclusions and Future Prospects

Plants possess the ability to manufacture a diverse range of chemical compounds, which exhibit potential significance in various biological contexts. Numerous phytochemicals have the potential to effectively address various human illnesses and enhance long-term well-being. [15,29]. Neurodegenerative diseases and cancer are of significant concern in the medical field due to the absence of efficacious therapeutic interventions and the alarming increase in the number of individuals affected by these conditions. Despite the varied clinical presentations of various illnesses, they all exhibit a shared cellular stress response. The cellular stress responses encompass various physiological processes, such as oxidative stress and inflammation. In recent years, there has been a significant focus on the potential of small molecules, specifically flavonoids, to mitigate cellular stress [130].

**Figure 4:**
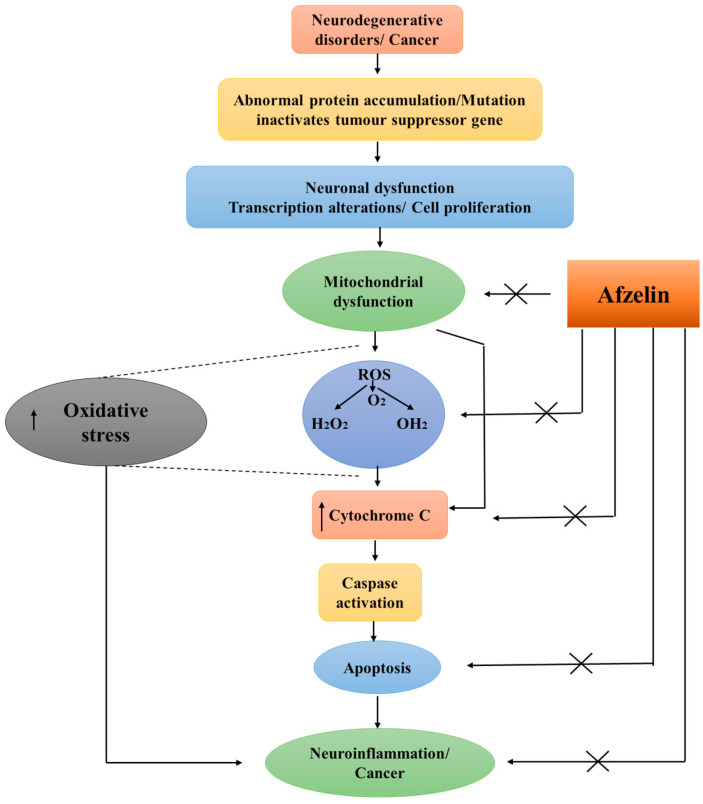
Therapeutic potential of afzelin in neurodegenerative diseases.

Afzelin (kaempferol 3-O-rhamnoside) is a representative of the flavonoid class of compounds. It has been observed to occur in a diverse range of plant sources. The potential benefits it may give to human health are beginning to be recognized. Afzelin demonstrates a wide array of biological activities, such as antioxidative, anti-inflammatory, anti-cancer, and neuroprotective properties. These characteristics make it a promising candidate for the prevention and treatment of neurodegenerative diseases that are characterized by oxidative stress and disruptions in cell death pathways (Figure 4).

The utilization of botanical extracts and isolated compounds in the management of neurodegenerative disorders and cancers encompasses diverse methodologies and presents inherent advantages and drawbacks. Plant extracts frequently consist of intricate combinations of diverse chemicals that are present in a given plant. These extracts have the potential to encompass a diverse array of phytochemicals, including polyphenols, alkaloids, and flavonoids. The intricate nature of these extracts presents difficulty in precisely determining the mechanisms of action and distinguishing the specific components accountable for therapeutic benefits. On the contrary, the obtaining of isolated substances involves the extraction and purification of particular components from plants. This methodology enables the examination of specific compounds and their impact on disorders, hence facilitating the identification of the exact bioactive component responsible for the therapeutic outcome. Furthermore, plant extracts contain substances that can increase the bioavailability of active ingredients or demonstrate synergistic effects. Nevertheless, the existence of additional chemicals may also potentially impede the processes of absorption, distribution, metabolism, and excretion (ADME) of bioactive constituents. In contrast, isolated substances frequently exhibit distinct pharmacokinetic characteristics, facilitating a comprehension process of ADME inside the human body. Understanding this information is of utmost importance when determining appropriate dose and treatment approaches. The safety characteristics of plant extracts can differ based on their specific composition. They may contain chemicals that have the potential to induce adverse effects or interact with other pharmaceutical substances. Isolated chemicals exhibit a greater degree of predictability in terms of safety. Nevertheless, it is important to note that even isolated substances can potentially exhibit adverse consequences. The ADME and toxicity profiling of afzelin and various plant extracts containing this flavonoid has not been performed (except for genotoxic studies) and constitutes one of the remaining issues that will determine the therapeutic usefulness of this compound. Furthermore, its blood–brain barrier (BBB) permeability has not been examined. The BBB serves as a crucial protective factor that effectively limits the permeability of medicines and therapeutic agents, hence impeding their access to the brain. The development of pharmaceutical agents capable of efficiently traversing the BBB to achieve precise localization within CNS is a major challenge. Strategies aimed at improving the bioavailability of these substances, such as the utilization of nanoformulations or prodrugs, have the potential to increase their pharmacokinetic characteristics and ultimately optimize their therapeutic efficacy. 

The diverse composition of plant extracts may provide a wider range of activity, rendering them appropriate for addressing various targets in the context of neurodegenerative disorders. This can confer advantages in the context of diseases characterized by complex pathologies. In the context of therapeutic interventions, the utilization of isolated chemicals enables the precise targeting of a single molecular target or pathway. This approach is advantageous in cases where a comprehensive understanding of the illness mechanism exists and necessitates a highly focused strategy. 

Afzelin has been shown to elicit a complex cellular response involving its suppressive effects on NF-κB or MAPK pathway conferring reduced expression of pro-inflammatory mediators or down-regulation of NO synthesis, inhibition of tyrosinase activity or up-regulation of molecular signaling pathways involving CREB-BDNF or influence on mitochondrial quality control and function. Moreover, it showed significant oxidative stress mitigating effects associated with ROS scavenging, metal chelation, and the influence of the NRF2 signaling pathway. 

In the context of neurodegenerative diseases, current therapies often focus on symptomatic management rather than addressing the underlying disease mechanisms. For example, in Alzheimer’s disease, acetylcholinesterase inhibitors such as donepezil and memantine are commonly prescribed to alleviate cognitive symptoms, but they do not halt disease progression [131]. Similarly, in Parkinson’s disease, dopamine replacement therapy with levodopa is a mainstay treatment for managing motor symptoms, but it does not prevent the degeneration of dopaminergic neurons [132]. In contrast, afzelin offers a multifaceted approach to addressing neurodegenerative diseases by targeting oxidative stress, inflammation, mitochondrial dysfunction, and neuroprotection, as outlined in the manuscript. Its ability to modulate redox signaling pathways, scavenge free radicals, and inhibit inflammatory mediators suggests a potential neuroprotective effect that may complement existing therapies. According to a recent study of afzelin in Parkinson’s disease, the cataleptic behaviors and locomotor activity of rats were greatly improved when administered with a combination of reserpine at a dose of 1 mg/kg and afzelin at doses of 5 mg/kg, 10 mg/kg, and 20 mg/kg. Furthermore, the administration of afzelin alone resulted in the induction of BCL-2 expression, suggesting potential neuroprotective characteristics. This study yielded valuable insights into the efficacy of afzelin in managing catalepsy and other degenerative neurological illnesses. Therefore, further investigations are required to ascertain the causes of the reactions and ascertain the enduring impacts of Afzelin on these situations [133].

Similarly, in the context of cancer, current treatments such as chemotherapy, radiation therapy, and targeted therapies often have significant side effects and may lead to drug resistance [134,135]. Afzelin’s anti-cancer properties, including its ability to induce apoptosis and inhibit inflammatory pathways, offer a novel approach to cancer treatment that may have fewer adverse effects compared to conventional therapies and may help prevent the development of treatment resistance or constitute a novel scaffold for drug discovery endeavors.

Furthermore, Wu et al. presented a method that is both ecologically sustainable and efficient in terms of speed and productivity for the extraction and purification of four bioactive components from *Zanthoxylum bungeanum* leaves, including afzelin. The findings can be employed for the incorporation of *Z. bungeanum* leaves as a dietary supplement in an industrial context, offering an accessible source of afzelin for therapeutical use [136]. Similarly, de Sousa et al. provided a validated chromatographic method for the detection of afzelin in *Copaifera langsdorffii* extracts by HPLC, providing another method suitable for the detection and isolation of this flavonoid [137]. The process of standardizing plant extracts to assure constant quantities of bioactive chemicals is of particular importance as it might present challenges, primarily stemming from inherent variances in plant material. Quality control can be more difficult compared to isolated substances. Isolated compounds can be synthesized or purified to a high degree of purity, enabling precise dosing and consistent therapeutic effects. Furthermore, the ease and cost-effectiveness of the afzelin isolation and purification procedure are of importance. In order to provide care to a diverse patient population, it is imperative that the treatment is made readily available and economically feasible, taking into account the socioeconomic obstacles encountered by those afflicted with neurodegenerative disorders and cancers.

In summary, the future prospects for utilizing plant-derived compounds like afzelin in the treatment of neurodegenerative diseases and cancer are promising. Afzelin, a flavonoid with multifaceted properties, holds potential as a therapeutic agent due to its anti-inflammatory, antioxidative, anti-cancer, and neuroprotective effects. Additionally, recent advancements in extraction and purification methods, such as those demonstrated by Wu et al. and de Sousa et al., offer sustainable and efficient means of obtaining afzelin and other bioactive compounds from natural sources. While challenges remain, including standardization of plant extracts and addressing bioavailability and BBB issues, the continued exploration of plant-derived compounds, including afzelin, represents a valuable avenue for developing novel treatments for neurodegenerative diseases. 

While this paper extensively covers in vitro and animal studies, it is crucial to acknowledge the necessity of transitioning these findings into clinical settings to ascertain the efficacy and safety of afzelin in humans. As of the current literature review, there are no clinical trials or human studies available for afzelin. Therefore, the focus of this review paper has been on detailing the results from preclinical studies, which provide valuable insights into the potential mechanisms of action and therapeutic effects of afzelin.

## Abbreviations

Aβ	amyloid-beta
AADC	aromatic L-amino acid decarboxylase
ACH	acetylcholine
AChE	acetylcholinesterase
ADMET	absorption, distribution, metabolism, excretion, and toxicity
ALS	amyotrophic lateral sclerosis
AR	aldose reductase
ARE	antioxidant response element
ATP	adenosine triphosphate
BBB	blood–brain barrier
BCL2	B-cell lymphoma 2
BDNF	brain-derived neurotrophic factor
CLE	hydroalcoholic extract of *Copaifera lansdorffii* Desf.
CNS	central nervous system
CO	carbon monoxide
CREB	cyclic AMP response element-binding protein
DAQ	dopamine quinone
DNM1L	dynamin-related protein 1
DQ	DOPAquinone
ERK	extracellular signal-regulated kinase
FAK	focal adhesion kinase
FHF	fulminant hepatic failure
FIR	far-infrared
FTD	frontotemporal dementia
GalN	D-galactosamine
GSK-3β	glycogen synthase kinase-3β
GSTs	glutathione S-transferases
HMGB1	high-mobility group box 1
HO-1/HO-2	heme oxygenase-1/2
HPLC	high-performance liquid chromatography
IL-1/IL-6	interleukin-1/6
iNOS	inducible nitric oxide synthase
JNK	c-Jun N-terminal kinase
LC	locus coeruleus
LC-MS/MS	liquid chromatography–tandem mass spectrometry
LIMK1	LIM domain kinase 1
LPS	lipopolysaccharide
MAPK	mitogen-activated protein kinase
MFN2	mitofusin 2
MRCKα	myotonic dystrophy kinase-related Cdc42-binding kinase α
MS	multiple sclerosis
MtDNA	mitochondrial DNA
NF-κB	nuclear factor kappa B
NM	neuromelanin
NQO1	NAD(P)H quinone dehydrogenase 1
NRF1	nuclear respiratory factor 1
NRF2	nuclear factor erythroid 2-related factor 2
OMM	outer mitochondrial membrane
P75NTR	P75 neurotrophin receptor
PARKIN	parkin protein
pFAK	phosphorylated focal adhesion kinase
PGC-1α	PPAR-γ coactivator 1α
PI3K	phosphatidylinositol 3-kinase
PINK1	PTEN-induced putative kinase 1
PMEE	*Polygonum minus* ethanolic extract
RAC1	Ras-related C3 botulinum toxin substrate 1
ROCK1	Rho-associated coiled-coil-containing protein kinase
ROS	reactive oxygen species
SOD1/SOD2	superoxide dismutase 1/2
SN	substantia nigra
TFAM	mitochondrial transcription factor A
TH	tyrosine hydroxylase
TNBC	triple-negative breast cancer
TNF-α	tumor necrosis factor-alpha
TRKB	tropomyosin kinase B
